# Odontoblasts in Equine Hypsodont Teeth—How They Cope with Permanent Occlusal Wear

**DOI:** 10.3390/ani16020341

**Published:** 2026-01-22

**Authors:** Laura Beate Heilen, Jessica Roßgardt, Jutta Dern-Wieloch, Jörg Vogelsberg, Carsten Staszyk

**Affiliations:** Institute of Veterinary-Anatomy, -Histology and -Embryology, Faculty of Veterinary Medicine, Justus-Liebig-University, 35392 Giessen, Germany; jessica.rossgardt@vetmed.uni-giessen.de (J.R.);

**Keywords:** equine dentistry, endodontium, odontoblasts, hypsodont teeth

## Abstract

Horses’ hypsodont (high-crowned) teeth face permanent dental wear. This is compensated for by a continuous eruption, which requires a high adaptability of odontoblasts; otherwise, the dental pulp would be exposed. Here, we report on how equine odontoblasts respond to the challenge of maintaining a high production rate of dentin. We analyzed CD90, a marker of odontoblastic differentiation, and nestin, a marker of mature odontoblasts, in equine pulpal tissue via immunofluorescence. For comparison, we examined the hypselodont (ever-growing) incisors and brachydont (short-crowned) molars of rats. Immunofluorescence and Western blot analysis of pulpal tissue revealed a higher content of CD90-positive cells in hypsodont equine teeth than in brachydont and hypselodont rat teeth. The odontoblastic layer of hypsodont teeth was positive for CD90 (marker for differentiating odontoblasts), which was not the case for brachydont and hypselodont rat teeth. Most samples of hypsodont teeth were negative for nestin, whereas in hypselodont and brachydont teeth, odontoblasts were positive for nestin (marker for mature odontoblasts). Our findings suggest that there is a constant replacement of odontoblasts in the equine dentition, enabling a continuous high production rate of dentin. These results contradict the idea of lifelong vital, postmitotic and productive odontoblasts.

## Simple Summary

In short-crowned teeth, such as human teeth, the tooth surface is sealed by enamel. The underlying dentin is produced by odontoblasts. Odontoblasts are known as postmitotic cells that produce relatively small amounts of dentin continuously throughout the tooth’s lifespan. However, equine high-crowned teeth are exposed to massive dental wear due to their abrasive diet. This is compensated for by the constant eruption of the teeth. In equines, the dentin is exposed to the tooth surface, so the pulp cavity underneath the dentin is permanently at risk of being opened. Thus, one might ask how odontoblasts compensate for this loss, given that they produce only small amounts of dentin in short-crowned teeth. We discovered that equine odontoblasts express CD90, a marker typically found in immature cells. Therefore, we assume that odontoblasts, which are only replaced after major damage in short-crowned teeth, must undergo continuous renewal in high-crowned equine teeth.

## 1. Introduction

In equine teeth, odontoblasts must compensate for the continuous loss of dentin caused by permanent occlusal wear.

**Figure 1:**
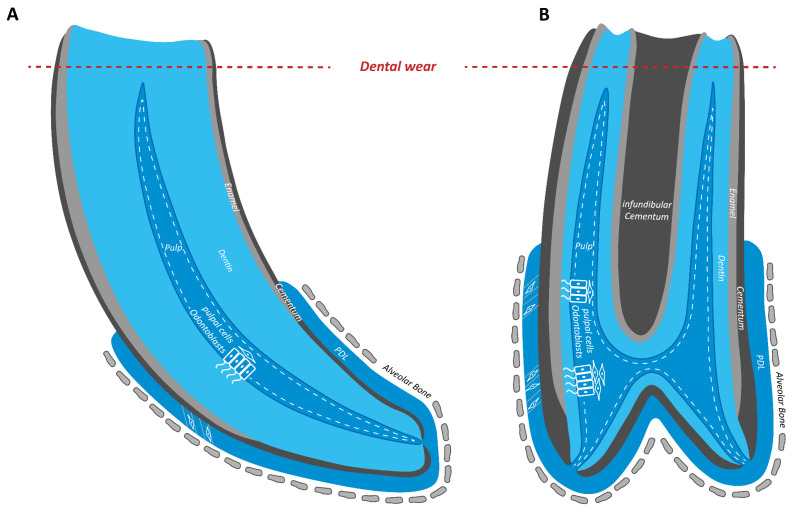
Scheme of an equine hypsodont incisor (**A**) and cheek tooth (**B**) exposed to dental wear (red dotted line). PDL: Periodontal Ligament.

Naturally, the equine diet primarily consists of silicate-enriched grasses contaminated with grit and soil, which leads to permanent dental wear. This is compensated for by constant eruption of the hypsodont (high-crowned) teeth [1,2]. Nevertheless, without the persistent production of occluding dentin by odontoblasts, the dental pulp would be exposed (Figure 1).

**Figure 2:**
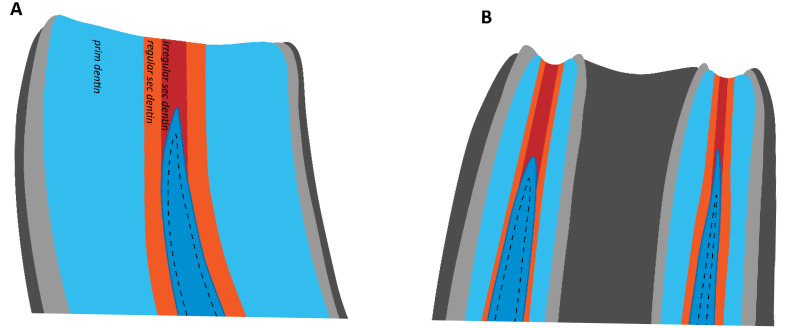
Different types of dentin in an equine incisor (**A**) and cheek tooth (**B**).

Thus, the occlusal surface is constantly sealed by regular secondary and irregular secondary dentin. Figure 2 shows that regular secondary dentin is produced circumpulpally, while irregular secondary dentin is deposited occlusal to the pulp horns’ cusps [3,4,5]. Regarding its structure, irregular secondary dentin resembles tertiary dentin in brachydont teeth. Tertiary dentin is only produced in response to stimuli such as trauma, caries, or restorative processes [4]. However, because irregular secondary dentin in hypsodont teeth is produced in reaction to a physiological stimulus, it should not be termed tertiary dentin [5].

Due to the constant adjustment of the occlusal surface in hypsodont teeth, odontoblasts must be much more productive than those in brachydont teeth. Odontoblasts are generally described as postmitotic cells that are not replaced during the lifespan of a healthy tooth [6,7,8]. During dental development, odontoblasts originate from migrating neural crest cells that differentiate upon interacting with the enamel organ epithelium and its basement membrane [7,9,10,11]. The enamel organ originates from the primitive oral epithelium and is responsible for enamel formation. A complex signaling network consisting of different paracrine molecules, such as transforming growth factor β, modulates odontoblastic differentiation [12]. Once tooth development is complete and the enamel organ has disappeared, differentiation of new original odontoblasts is no longer possible. After mild pathological stimuli, surviving odontoblasts can secrete a tertiary dentin, also known as reactionary dentin. When original postmitotic odontoblasts are irreversibly damaged, they are replaced by odontoblast-like cells that produce a special type of tertiary dentin called reparative dentin. However, it is impossible to distinguish between the two types of tertiary dentin based on morphological characteristics [13,14].

Secretory-active odontoblasts are columnar-shaped, highly polarized cells with a pseudostratified, epithelioid organization [15,16]. They possess one primary cytoplasmic process, also known as “Tomes fiber”, which is formed when dentin production begins. This process represents the secretory pole and elongates as dentin is deposited around it. It mainly consists of microtubules and actin filaments [11,17]. Lateral branches of the primary process form a network inside the mineralized dentin matrix. Thus, there is a direct connection between the dental pulp and the dentin, forming a structural and functional unit referred to as the endodontium [18].

Furthermore, odontoblasts undergo an aging process. In humans, primary dentin is secreted at a rate of 4–8 µm per day, while the production of secondary dentin decreases to a rate of approximately 0.5 µm per day [15,19]. This reduction is accompanied by changes in odontoblast morphology. The mature odontoblast stage is characterized by autophagic activity and basally located organelles. As odontoblasts age, their cell size appears reduced and their shape becomes flatter [16,20].

Despite these characteristics, equine odontoblasts must produce large amounts of subocclusal dentin throughout their lifetime to compensate for the 3–4 mm of occlusal wear per year [21,22]. Based on this occlusal loss, one can calculate a reproduction rate of approximately 8–10 µm per day, corresponding to non-erupted brachydont teeth [16,19]. Thus, one must ask whether equine odontoblasts retain a high level of productivity throughout their lives or whether they undergo constant cellular remodeling to ensure this high production rate of secondary dentin.

To answer this question, we evaluated the presence of CD90 and Nestin in equine dental pulp and compared the results to pulp tissue from the hypselodont incisors and brachydont molars of rats.

CD90 is known as a marker for mesenchymal stem cells (MSCs) and is also expressed by dental pulp stem cells (DPSCs). DPSCs can replace damaged odontoblasts and form odontoblast-like cells that produce reparative dentin [23,24]. In brachydont teeth, CD90-positive cells are typically located in the subodontoblastic layer [25,26].

Nestin, an intermediate filament, plays a role in nerve and muscle development [27,28]. Terling et al. [29] discovered that nestin is also expressed during tooth development in rodents and can be used as a marker for mature odontoblasts. During tooth development, nestin expression progressively becomes restricted to odontoblasts, and it is not downregulated in adult, functional odontoblasts [30].

It is unknown whether the population of secretory-active odontoblasts in equine hypsodont teeth is composed of mature, postmitotic, nestin-positive odontoblasts or whether it is continuously replaced by newly differentiated odontoblast-like cells. Furthermore, little is known about a subodontoblastic reservoir of CD90-positive cells that may differentiate into odontoblast-like cells to replace old or damaged odontoblasts.

## 2. Materials and Methods

### 2.1. Donors

Equine teeth and tissues were obtained from four horses and one pony that were slaughtered by a commercial butcher via captive bolt followed by bleeding for reasons unrelated to this study. Therefore, no ethical approval was required. The related kTV number provided by the regional council is 19 c 20 15 h 02 Gi 18/17 kTV 5/2021. The donors were between 5 and 12 years old.

The rat tissues originated from an in-house breeding colony with parents from Charles River WIGA (Sulzfeld, Germany) at the Institute of Veterinary Physiology and Biochemistry at the Justus Liebig University Giessen. All rat tissues used in this study were tissue remnants remaining after previous studies at the Institute of Veterinary Physiology and Biochemistry. The studies were performed according to the German Law on Animal Welfare, registered at the regional council of Hesse, Germany, and authorized by the Justus Liebig University Giessen (approval numbers GI 799_M and GI 800_M). Details of the donors are listed in Table 1.

### 2.2. Preparation of Equine Hypsodont Incisors and Cheek Teeth

Tissue preparation was carried out with a few modifications, as previously described by Heilen et al. [31]. Briefly, the heads were skinned and fleshed, and the maxillae and mandibles were trimmed with a water-cooled band saw. The Teeth were identified according to Triadan’s [32] and Floyd’s [33] tooth numbering system. After macroscopically examining the teeth to select donors with clinically unremarkable teeth, the incisor and cheek tooth arches were separated and further processed using a diamond-coated, water-cooled micro-band saw (MBS 240/E; Proxxon S.A., Wecker, Luxembourg).

One cheek tooth per donor was selected and removed from the dental arcade through incisions. Afterwards, the tooth was cut into horizontal sections approximately 0.8 mm thick, from occlusal to apical. The sequence of the horizontal sections was assigned by marking them with vertical saw kerfs through the dentin.

One quadrant, including incisors 01 to 03, was selected and separated by a midline cut mesial to 01. The incisor arcades were sectioned into approximately 0.8 mm thick slices from occlusal to apical. The sequence was also marked with vertical saw kerfs. After sectioning, all specimens were fixed in 10% buffered formalin (pH 7) and preserved for histological processing.

**Figure 3:**
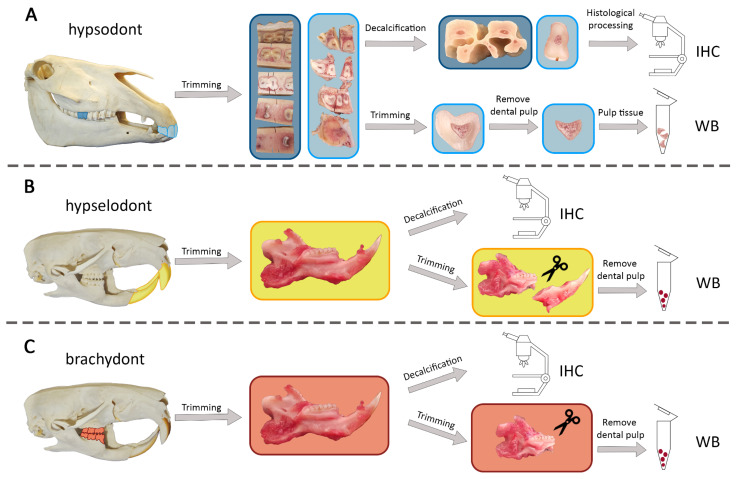
Sample preparation for immunofluorescence and Western blot. (**A**) Preparation of hypsosdont teeth. The incisors and one molar (209) are marked in two different shades of blue. (**B**) Preparation of hypselodont teeth. The hypselodont incisors are highlighted in yellow. (**C**) Preparation of brachydont teeth. The brachydont molars are highlighted in orange.

The dental pulp tissue of donors 3 to 5 was isolated from the incisors using another incisor arcade that included incisors 01 to 03. The dental pulp was pulled out of the pulp cavity of 0.8 mm thick transverse slices via Hedstrom files. All tissue samples were cryopreserved at −150 °C for future use. Figure 3A illustrates the entire procedure.

### 2.3. Preparation of Rat Hypselodont Incisors and Brachydont Molars

To obtain the upper and lower teeth, the heads were separated, skinned, and fleshed. Then, the mandible was separated and cut median into halves. For subsequent histological processing, one half was fixed in 10% buffered formalin (pH 7) for 24 h. The other half was cut transversely caudal to the molars using a diamond-coated, water-cooled micro-band saw. During this procedure, the pulp cavity of the incisor was opened, and the dental pulp was removed with forceps. To isolate the dental pulp from the cheek teeth, the dental crown was cracked using rongeur forceps. Small amounts of dental pulp were then obtained under a stereo magnifier with a 24-gauge cannula. Pulpal tissue from different cheek teeth was pooled to increase the volume. All tissue samples were cryopreserved at −150 °C for further use.

A transversal cut was made caudal to the molars to separate the maxilla. Then, the incisors were separated. The incisors and cheek teeth were fixed in 10% buffered formalin (pH 7) for 24 h and stored for histological processing. This procedure is illustrated in Figure 3B,C.

### 2.4. Immunofluorescence of Histological Sections from Dental Pulp

Immunofluorescence for CD90 and nestin was performed with a few modifications, as previously described by Heilen et al. [31].

**Figure 4:**
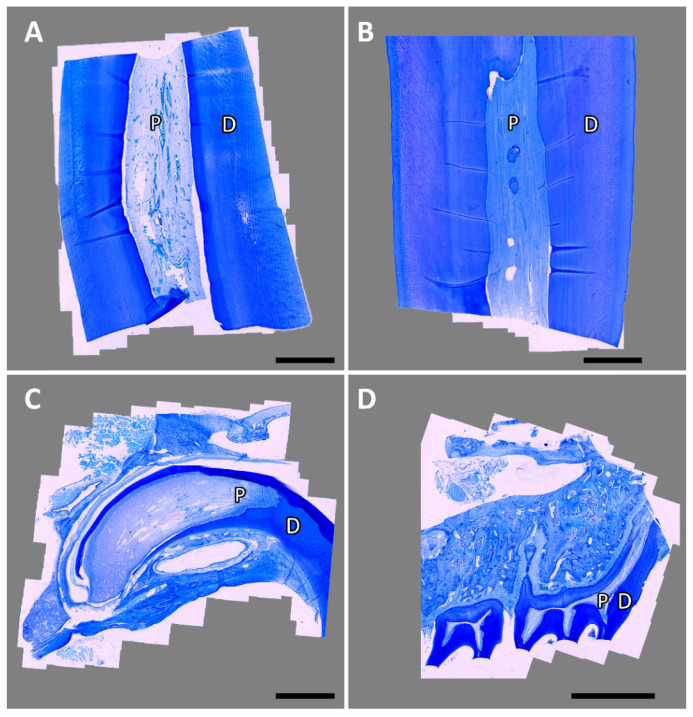
Scanned toluidine blue-stained sections. Dental pulp (P) and surrounding dentin (D) of an equine hypsodont incisor (**A**), an equine hypsodont cheek tooth (**B**), a rat hypselodont incisor (**C**) and rat brachydont molars (**D**). The scale bar represents 2 mm.

Initially, the fixed specimens were washed and decalcified on a platform shaker (Polymax 1040; Heidolph Instruments, Schwabach, Germany) in a solution of buffered ethylenediaminetetraacetic acid. This process took 2 weeks for rat specimens and up to 10 weeks for equine specimens. Next, the equine specimens were trimmed with a scalpel to leave only the pulp tissue and surrounding dentin. All specimens were transferred to embedding cassettes (Simport™ Acetal Macrosette; Fischer Scientific GmbH, Schwerte, Germany). The equine samples were then decalcified for another 2 weeks. The samples were paraffin-embedded, sectioned, and stained with toluidine blue according to Roßgardt et al. [34]. Toluidine blue-stained sections were evaluated by light microscopy (Leica DM2500, camera: DMC4500; Leica Microsystems GmbH, Wetzlar, Germany) and partially scanned via an automated picture-aligning tool (Leica LAS XY Live Image Builder; Leica Microsystems GmbH). Examples are shown in Figure 4. Next, slices with intact tissue were selected and prepared for immunohistochemistry as previously described by Heilen et al. [31]. A list of the applied primary and secondary antibodies is provided in Table 2. As a negative control, the samples were incubated only with the secondary antibody. Finally, the examination was performed using a Zeiss Axio Observer Z.1 (Carl Zeiss, Göttingen, Germany).

### 2.5. Evaluation of Immunofluorescence

For every equine donor, at least one incisor and one cheek tooth were analyzed. Care was taken that at minimum, one sample of the occlusal, middle, and apical area was evaluated.

**Figure 5:**
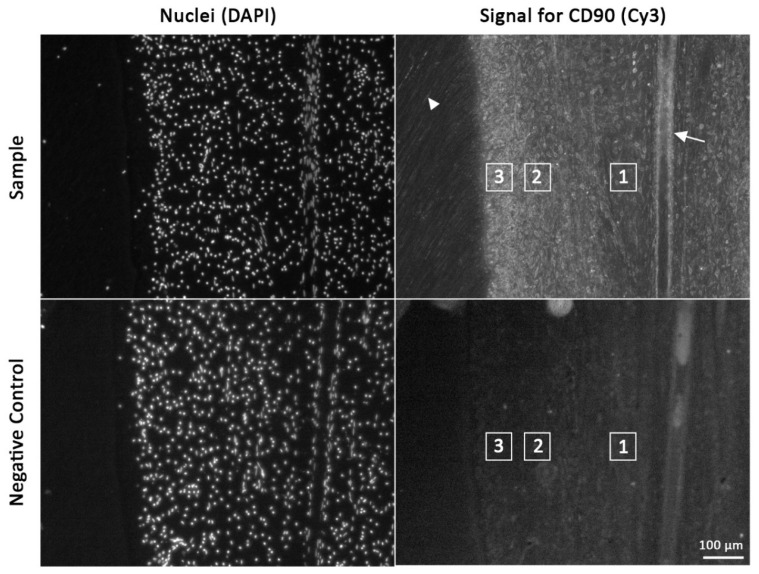
Evaluation of immunofluorescence for CD90 in samples of equine dental pulp. The regions of interest are highlighted with a numbered rectangle. 1: pulp core. 2 subodontoblastic layer. 3: odontoblastic layer. CD90-positive odontoblastic processes are marked with an arrowhead and CD90-positive pericytes of a blood vessel with an arrow.

Each sample was evaluated according to a three-step protocol. First, the presence of cells with a positive signal was examined. Then, the position of the positive signal was identified. Three zones were defined for this purpose: one in the odontoblastic layer, one in the underlying subodontoblastic layer, and one in the pulp core. Finally, the signal intensities of these three zones were compared with each other (Figure 5). Additionally, positive signals inside odontoblastic processes and in the perivascular area were documented.

Rat incisor and cheek tooth samples were used for comparison. The same procedure was applied to these samples. To exclude autofluorescence of the tissue or nonspecific binding of the secondary antibody, a negative control was available for each sample.

### 2.6. Western Blot

To validate the content of CD90 in pulp tissue from different types of teeth, a Western blot was performed.

The cryopreserved tissue was transferred to a loading buffer and denatured for 5 min at 95 °C. For gel electrophoresis, 15 µL of sample was loaded into each gel pocket of a 4–12% Bis-Tris gel (Blot™, NW04122BOX; Thermo Fisher, Waltham, MA, USA). As a marker, 5 µL of a prestained protein ladder (PageRuler™; Thermo Fisher, 26619) was applied to one gel pocket. The gel electrophoresis ran for 30 min at 200 V in an XCELL SureLock™ Mini-Cell electrophoretic system (Thermo Fisher, EI0001). NuPage™ MES SSDS buffer (Thermo Fisher, NP0002) was used for electrophoresis. Then, the gel was transferred onto a nitrocellulose membrane (Invitrogen™, Thermo Fisher, LC2009) for 70 min at 30 V. For total protein staining, Revert 700 Total Protein Stain (LI-COR, 926-11011, Lincoln, NE, USA) was applied for 5 min, after which it was washed out twice, each time for 30 s, with Revert Wash Solution (LI-COR, 926-11012). The membrane was scanned at 700 nm using a LI-COR Odyssey 9120 with IS Image Studio Version 5.2 software. For blocking, the membrane was incubated in 5% skim milk for 60 min at room temperature. Then, the primary antibody (Table 3) was applied overnight at 4 °C. Finally, the membrane was incubated with the secondary antibody (Table 3) for 1 h and scanned using the LI-COR Odyssey 9120 at 800 nm.

Quantification was performed using Empiria Studio^®^ 2.2 Software (LI-COR). The pooled samples of rat molars ran on seperate gels together with rat brain tissue that was used as a positive control and standard. The results were presented using GraphPad Prism 6.

## 3. Results

### 3.1. Immunofluorescence of CD90 in Dental Pulp Section of Equines and Rats

In equine hypsodont teeth, CD90-positive cells were found in all regions of interest, from occlusal to apical. Remarkably, in the occlusal part the CD90 intensity decreased from the odontoblastic layer to the pulp core in all teeth (incisors and cheek teeth) (Figure 5). In the apical direction, 6 of 17 samples showed a shift in the highest signal intensity from the odontoblastic layer to the subodontoblastic layer and the pulp core.

**Figure 6:**
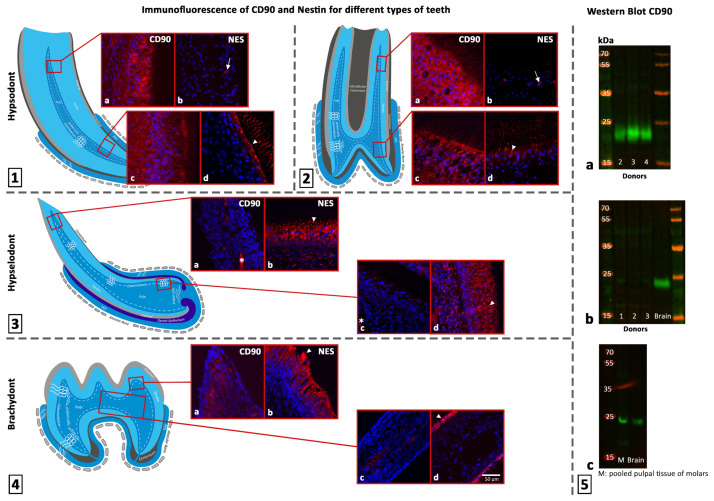
Overview of the results. (**1**–**4**) Immunofluorescence of CD90 and Nestin for different types of teeth. Each tooth type is represented by a scheme. The hypsodont tooth is present as an incisor (**1**) and a cheek tooth (**2**). The corresponding regions of interest for every tooth are highlighted with red rectangles in the occlusal and apical areas. The left picture shows the immunofluorescence for CD90, and the right picture for nestin, whereby nuclei are stained blue with DAPI. Arrowheads highlight the increased nestin signal at the apical pole of odontoblasts. Auto-fluorescent erythrocytes are indicated by asterisks. In hypsodont teeth, the CD90 signal was present in the subodontoblastic and odontoblastic layer (**1a**, **1c**, **2a**, **2c**). Not all odontoblasts showed a signal for nestin. The signal was decreasing towards occlusal, with only some positive cells highlighted by arrows (**1b**, **1d, 2b, 2d**). In hypselodont teeth, a CD90 signal was barely detectable (**3a**, **3c**). Odontoblasts and the subodontoblastic layer of hypselodont teeth expressed nestin (**3b**, **3d**). In the brachydont tooth, CD90 was expressed in the subodontoblastic layer of the coronal pulp (**4a**). The subodontoblastic layer of the root pulp was negative for CD90 (**4c**). Odontoblasts expressed nestin (**4b**, **4d**). (**5**) Results of CD90 Western blot for the different tooth types. Full scans of all gels, including those stained with total protein, are provided in the Supplementary Materials (Figures S1–S4). (**5a**) Pulpal tissue from hypsodont equine teeth (donors 2–4) showed strong bands at 22 kDa. (**5b**) In pulpal tissue from hypselodont rat incisors, the signal was nearly undetectable. (**5c**) Pooled pulpal tissue from rat brachydont molars showed a signal at 22 kDa. It ran on a separate gel with rat brain tissue as positive control.

The CD90 signal in the perivascular area was inconsistent in all samples. Some blood vessels showed a positive signal for CD90, while others did not. Numerous odontoblastic processes showed a positive CD90 signal (Figure 5 and Figure 6).

Apart from the blood vessels, CD90-positive cells were only detected in the subodontoblastic area of the coronal pulp of rat brachydont molars. The odontoblastic layer and cells in the central area of the dental pulp were negative for CD90. As with hypsodont teeth, some blood vessels showed a positive signal for CD90, and some did not.

In the hypselodont incisors of rats, CD90-positive cells were only detected perivascularly in the large blood vessel system underlying the odontoblastic layer. All other cells did not show a CD90 signal.

To summarize, the equine hypsodont tooth showed the strongest CD90 signal. Neglecting the perivascular area, the signal in brachydont rat molars was limited to the subodontoblastic area of the coronal pulp. In hypselodont rat incisors, no signal was detected in any of the defined areas (see Table 4). Erythrocytes showed autofluorescence in all samples. The results for CD90 are illustrated in Figure 6.

### 3.2. Western Blot of CD90

**Figure 7:**
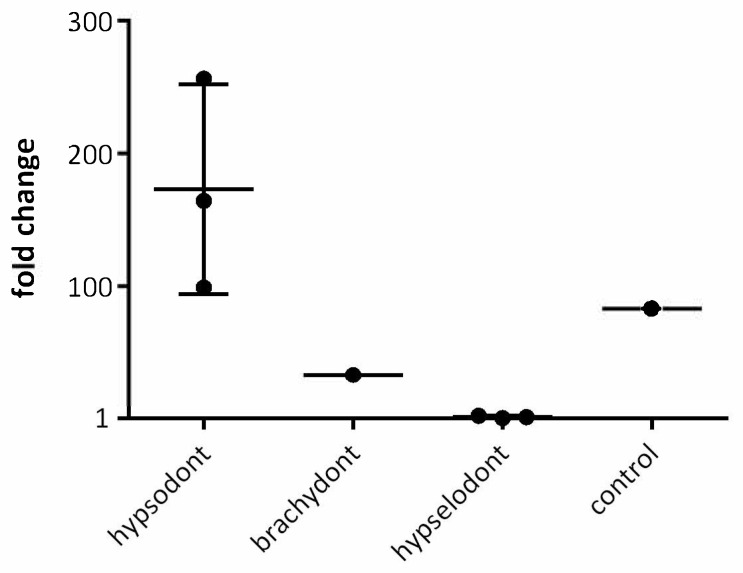
Fold Change Chart of CD90-Western blot. As a positive control rat brain tissue was used. For better illustration, the mean value of hypselodont samples was set to 1 and compared to the other samples.

The results of the CD90 immunofluorescence assay were validated by Western blot. Therefore, samples of each type of tooth were analyzed. Figure 6 shows that equine pulpal tissue from donors 2–4 exhibited a strong band at 22 kDa, which is characteristic of CD90. By contrast, pulpal tissue from rat incisors produced an almost undetectable signal. Pooled pulpal tissue from rat brachydont molars also showed a band at 22 kDa (see Figure 6). Quantifying the signals with total protein staining revealed that equine hypsodont teeth had the highest CD90 content by far, averaging 173-fold higher than hypselodont incisors. The CD90 content in rat brachydont molars was 32.76-fold higher than in hypselodont incisors, placing it between the other two types of pulpal tissue. These results align with those of the immunofluorescence for CD90. The fold changes are charted in Figure 7. Full scans of all gels, including those stained with total protein, are provided in the Supplementary Materials (Figures S1–S4).

### 3.3. Immunofluorescence of Nestin in Dental Pulp Sections of Equines and Rats

In equine hypsodont incisors and cheek teeth, nestin-positive cells were only present in the odontoblastic and subodontoblastic layers. The odontoblastic layer exhibited higher intensity. Donor 3 did not show any signal, whereas donor 1 showed nestin-positive cells from the occlusal to the apical region. Donors 2, 4, and 5 were mostly negative in the occlusal and middle positions. In odontoblasts, the nestin signal was very distinct at the apical pole and decreased toward the basal pole.

In rat brachydont molars, all odontoblasts showed a bright nestin signal, while all other cells were negative. A decrease in signal intensity from the apical to the basal pole was also observed in odontoblasts.

The results in rat hypselodont incisors were similar to those in rat brachydont molars, except for the subodontoblastic layer. There, cells with a diffuse nestin signal were found.

In summary, the equine hypsodont tooth showed the lowest nestin signal. In the brachydont molars and hypselodont incisors of rats, a signal was consistently present in the odontoblastic layer (Table 4). A detailed list of nestin-positive samples for each region can be found in Table S1 of the Supplementary Materials. All samples exhibited erythrocyte autofluorescence. The results for nestin are illustrated in Figure 6.

## 4. Discussion

Our results suggest that equine odontoblasts are continuously replaced by newly differentiated odontoblast-like cells. Most equine odontoblasts are positive for CD90, which is characteristic of DPSCs. DPSCs can replace damaged odontoblasts, differentiate into odontoblast-like cells, and produce reparative dentin [23,24]. Furthermore, a large subodontoblastic reservoir of CD90-positive cells forms a reserve for odontoblastic replacement.

### 4.1. Expression of CD90 in the Pulp Tissue of Different Types of Teeth

When we compared the expression of CD90 in the dental pulp of the three types of teeth, we found that hypsodont pulpal tissue had the highest expression by far. This was initially unexpected, because odontoblasts are generally considered postmitotic cells [35]. A closer look at brachydont teeth reveals that CD90-positive cells in the subodontoblastic layer differentiate into odontoblast-like cells following experimental mechanical stimulation, such as cavity preparation [26]. Furthermore, CD90 expression is higher in unerupted teeth than in erupted teeth [24,36,37]. These results suggest that CD90-positive cells play a role in the regeneration and differentiation of odontoblasts and odontoblast-like cells. In brachydont teeth, cells capable of replacing damaged odontoblasts are distributed differently, with an accumulation in the coronal pulp [38]. This is confirmed by our results and by Sano et al. [26]. CD90-positive cells are enriched in the coronal subodontoblastic layer but are absent in the subodontoblastic layer of the root pulp. Positive pericytes, however, are present in all areas. The coronal subodontoblastic location of CD90-positive cells facilitates the replacement of damaged odontoblasts after injury.

In equine hypsodont teeth, CD90-positive cells are distributed along the entire length of the tooth in the subodontoblastic layer. However, the different morphology of equine teeth must be taken into consideration. Because of their much longer dental crowns, a greater expansion of CD90-positive cells can be expected. Nevertheless, a CD90-positive subodontoblastic layer was also observed in the apical areas of the roots. This distribution of CD90-positive cells throughout the subodontoblastic layer of equine teeth may continuously provide new odontoblastic cells. Thus, the tooth can adapt continuously to eruption. Another difference in the hypsodont teeth is that odontoblasts express CD90. However, our results and the literature describe odontoblasts in unaffected brachydont teeth as differentiated, CD90-negative cells [25,26,37]. In damaged brachydont teeth, CD90-positive cells and other cells with mesenchymal stem cell markers can be observed in the odontoblastic layer due to accelerated differentiation and maturation [26,37]. This raises the question of why odontoblasts in equine teeth physiologically express CD90. Therefore, one should consider that these teeth must withstand permanent occlusal wear, and the occlusal dentin is always exposed to the oral cavity. Especially the latter, even if it is mild, can lead to inflammatory processes and may cause cell recruitment to replace affected odontoblasts [39]. In equine teeth, it seems that due to exposure of the endodontium [18] and permanent occlusal wear, a constant replacement of odontoblasts is initiated, to the extent that odontoblasts exhibit characteristics of mesenchymal stem cells. Furthermore, CD90-positive cells can be located in the pulp core, which has not been reported for brachydont teeth [25,26,37]. This demonstrates the extent of cell activation in equine hypsodont teeth. The shift in CD90 signal intensity from the odontoblastic layer to the subodontoblastic layer from occlusal to apical may be due to lower levels of cell activation in the apical region.

In our study, we included rat hypselodont incisors to reveal the differences in odontoblastic replacement between hypselodont and hypsodont teeth. Hosoya et al. [25] described CD90-positive cells in the subodontoblastic layer of rat incisors and proposed a mechanism similar to that of pulp regeneration in brachydont molars. However, An et al. [40] found that CD90-expressing cells were barely detectable in erupted adult mouse incisors and only regenerated during periods of rapid tooth growth, such as after incisor clipping. They showed that CD90-positive cells do not contribute to odontoblastic regeneration during normal growth rate homeostasis. They found CD90-expressing cells in the apical end, between the labial and lingual aspects of the cervical loop. Our results for CD90 in the hypselodont teeth showed only a faint perivascular signal. We could not detect a signal in the subodontoblastic layer or between the two aspects of the cervical loop. This may be due to the small number of CD90-positive cells in adult hypselodont rat incisors, which supports An et al.’s [40] hypothesis. In summary, it appears that two different mechanisms underlie tooth growth in hypselodont teeth. One mechanism is active during homeostasis, and the other is active when accelerated growth is necessary (for example, due to clipping). However, only the latter is dependent on CD90-expressing cells. Feng et al. [41] confirmed the hypothesis of a mesenchymal stem cell source near the cervical loop that contributes to odontoblastic replacement following damage. Furthermore, they discovered that pericytes also contribute to odontoblastic regeneration in small amounts.

All types of teeth have one thing in common: CD90-expressing cells play a role in odontoblastic replacement when the pulp–dentin complex is affected to a certain extent. Brachydont teeth do not require odontoblastic replacement unless there is damage, whereas hypsodont equine teeth face permanent occlusal damage due to constant dental wear. Therefore, they constantly replace odontoblasts and use the same mechanism as damaged brachydont teeth. Rats with hypselodont incisors are also exposed to permanent occlusal wear, but they depend on CD90-expressing cells only when the damage exceeds a certain degree that cannot be compensated for by the normal growth rate. This shows that regardless of tooth type, there is a conserved mechanism to compensate for the massive loss of dentin.

### 4.2. Expression of Nestin in Pulpal Tissue of Different Types of Teeth

Because nestin can be used as a marker for mature odontoblasts [29,30], it is suspected that all three types of teeth possess odontoblasts that express nestin. However, in samples of equine hypsodont teeth, cells in the odontoblastic layer often showed no nestin expression, although these cells had all the morphological characteristics of odontoblasts and must produce dentin; otherwise, the dental pulp would be affected. This may be explained by the extent of permanent occlusal wear, which is associated with the massive replacement of odontoblasts. This replacement leads to accelerated differentiation and maturation of juvenile odontoblasts that still possess characteristics of mesenchymal stem cells [26,37]. Another possibility is that replaced odontoblasts lose their capacity to express nestin. However, Sano et al. [26] describe recurring nestin expression in odontoblasts after damage, as well as co-localization of nestin and CD90.

Furthermore, if the odontoblastic layer expresses nestin, then the subodontoblastic layer also expresses nestin, albeit at a weaker level. This may be due to inclined sectioning or the differentiation process, which progresses from the subodontoblastic layer to the odontoblastic layer. A closer look at nestin expression in odontoblasts reveals that the signal moves toward the apical pole and odontoblastic extensions during differentiation [42]. This indicates that odontoblasts with a strong signal at the apical pole and inside their processes, as observed in our samples, are mature odontoblasts.

In the brachydont molars and hypselodont incisors of rats, odontoblasts expressed nestin, as confirmed by previous literature [29,30]. Our results also showed a signal in the subodontoblastic layer, possibly due to inclined sectioning. However, Quispe-Salcedo et al. [42] describe weak nestin expression in other pulpal cells. Furthermore, different authors [25,42,43] refer to a progression of odontoblastic differentiation from apical to occlusal in the hypselodont incisor. This progression demonstrates the normal growth rate of hypselodont incisors, which occurs apical from dental papilla cells adjacent to the inner enamel epithelium. As odontoblasts move closer to the incisal end, they elongate and mature until they reach an inactive, quiescent state [44]. This process is entirely different from that occurring after damage to hypselodont teeth. Lesions are not repaired by this mechanism.

## 5. Conclusions

**Figure 8:**
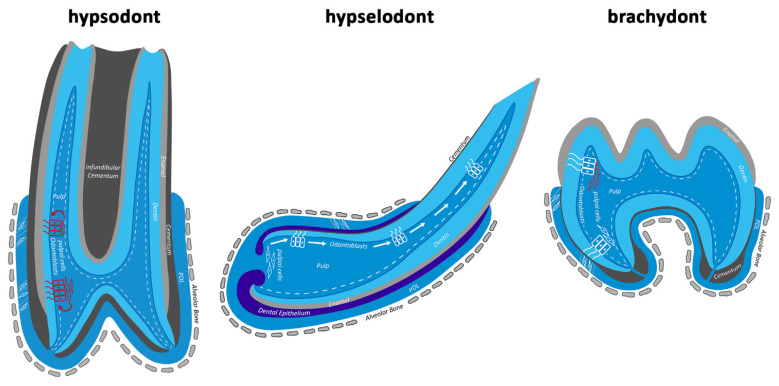
Odontoblasts in different types of teeth under physiological conditions. CD90-expressing cells are highlighted in red. In hypsodont teeth, odontoblasts are permanently replaced by CD90-positive cells from the subodontoblastic layer, placing the tooth in a “reparative status”. In hypselodont teeth, there is constant growth rate homeostasis, and new odontoblasts differentiate from apical up to the incisal end. This “regenerative status” is subject to a totally different mechanism, in which CD90-positive cells do not play a crucial role. In brachydont teeth, odontoblasts are not replaced under physiological conditions. They produce small amounts of secondary dentin and remain in a “resting status”. Following damage, odontoblastic replacement is limited but equivalent to that of a hypsodont tooth.

All types of teeth possess a repair mechanism associated with CD90-positive cells that exhibit characteristics of mesenchymal stem cells. Under physiological conditions, odontoblasts in brachydont teeth remain in a resting state, meaning they are not replaced and produce only small amounts of secondary dentin. In equine hypsodont teeth, odontoblasts appear to be permanently replaced by cells from the adjacent subodontoblastic layer. This facilitates the constant “repair” of the continuously worn occlusal surfaces. Thus, these teeth are in a “repair status” (Figure 8). In rats with hypselodont teeth, “post-odontoblasts” [44] at the incisal end are replaced by newly generated odontoblasts that migrate up from the apical bud. This means that these teeth are in a permanent “regenerating state” to maintain growth rate homeostasis [25,42,43]. The repair mechanism in hypselodont teeth only becomes active when growth rate homeostasis is disturbed due to lesions.

Equines do not possess such a mechanism to replace odontoblasts and create a constantly growing hypselodont tooth. During evolution, the habitat of equines changed from forested to open environments. This process was accompanied by a change in diet from the leaves of dicotyledonous plants to grasses contaminated with grit and soil, which caused massive dental wear. Thus, equines developed a method to adapt their primary brachydont teeth to these new conditions [45,46]. Therefore, a mechanism that was already present in brachydont teeth for compensation and repair of lesions after exposure of the pulp–dentin complex was converted. It remains open whether the findings presented here for the equine hypsodont dentition are also valid for other species featuring hypsodont teeth. As equine teeth are identified to show one of the highest hypsodonty indices [47,48], the demonstrated cellular mechanisms might represent an equine-specific adaptation to their high grade of hypsodonty. Accordingly, future studies are needed to answer the question whether the cellular adaptions found in equines can also be found in other hypsodont species and might be regarded as a general prerequisite to establish hypsodonty.

## Figures and Tables

**Table 1:** Details of Donors.

Horses				
No.	Age	Sex	Breed	Included Teeth
1	5 y	♀	Pony	101–103, 110
2	6 y	♀	Haflinger	309
3	10 y	♂	Warmblood	101–103, 409
4	12 y	♂	Hanoverian	101–103, 409
5	12 y	♀	Oldenburger	201–203, 308
**Rats**				
1	48 d	♀	Wistar	All teeth
2	49 d	♀	Wistar	All teeth
3	1 y	♀	Wistar	All teeth

**Table 2:** Antibodies for immunofluorescence.

Primary Antibodies			
Name	Manufacturer	Dilution	Application
Rabbit anti-mouse CD 90 Clone: D3V8A Cat No.: 13801	Cell signaling, Danvers, MA, USA	1:700	Equine, Rat
Rabbit anti-human Nestin Clone: aa1295-1344 Cat No.: LS-B5875	LSBio, Newark, CA, USA	1:200	Equine
Mouse anti-rat Nestin Clone: Rat-401 Cat No.: sc-33677	Santa Cruz, Dallas, TX, USA	1:100	Rat
**Secondary Polyclonal Antisera**			
**Name**	**Manufacturer**	**Dilution**	**Application**
Cy™3-conjugated AffiniPure^®^ donkey anti-rabbit, polyclonal IgG (H+L) (Code: 711-165-152)	Jackson ImmunoResearch, West Grove, PA, USA	1:400	Equine, Rat
Cy™3-conjugated AffiniPure^®^ goat anti-mouse, polyclonal IgG (Code: 115-165-164)	Jackson ImmunoResearch, West Grove, PA, USA	1:200	Rat

**Table 3:** Antibodies for Western Blot.

Primary Antibodies			
Name	Manufacturer	Dilution	Application
Rabbit anti-mouse CD 90 Clone: D3V8A Cat No.: 13801	Cell signalling, Danvers, MA, USA	1:1000	Equine, Rat
**Secondary Polyclonal Antisera**			
**Name**	**Manufacturer**	**Dilution**	**Application**
IRDye^®^ 800CW Goat anti- rabbit IgG Cat No.: 926-32211	LI-COR, Lincoln, NE, USA	1:10,000	Equine, Rat

**Table 4:** CD90 and NES immunofluorescence with neglection of perivascular CD90-signal.

	OD Layer		Subod-Layer		Pulp Core	
	CD90	NES	CD90	NES	CD90	NES
**hypsodont**	+	–/+	+	–/+	+	–
**hypselodont**	–	+	–	+	–	–
**brachydont**	–	+	+ *	–	–	–

−: negative, −/+: some samples negative and some positive, +: positive, * only in coronal pulp, Od: Odontoblastic, Subod: Subodontoblastic.

## Data Availability

The raw data supporting the conclusions of this article will be made available by the authors on request.

## Supplementary Materials

The following supporting information can be downloaded at: https://www.mdpi.com/article/10.3390/ani16020341/s1, Figure S1: Total protein of Figure 6(5a,5b); Figure S2: Original image of Figure 6(5a,5b); Figure S3: Total protein of Figure 6(5c); Figure S4: Original image of Figure 6(5c). Table S1: Percentage (%) of nestin-positive samples per region.
