# Supplementary material for: Odontoblasts in Equine Hypsodont Teeth—How They Cope with Permanent Occlusal Wear
Source: Animals (Basel). 2026 Jan 22;16(2):341. doi: 10.3390/ani16020341 (PMC12838237; doi:10.3390/ani16020341)
Supplement: Supplementary file 1 [file animals-16-00341-s001.zip › Table S1-nestin.pdf]

Table S1.: Percentage (%) of nestin positive samples per region

	OD Layer	Subod-Layer	Pulp Core
**Occlusal**	33,3%	22,2%	0%
**Middle**	22,2%	22,2%	0%
**Apical**	77,5%	44,4%	0%
