# Supplementary figures and images for: Odontoblasts in Equine Hypsodont Teeth—How They Cope with Permanent Occlusal Wear
Source: Animals (Basel). 2026 Jan 22;16(2):341. doi: 10.3390/ani16020341 (PMC12838237; doi:10.3390/ani16020341)

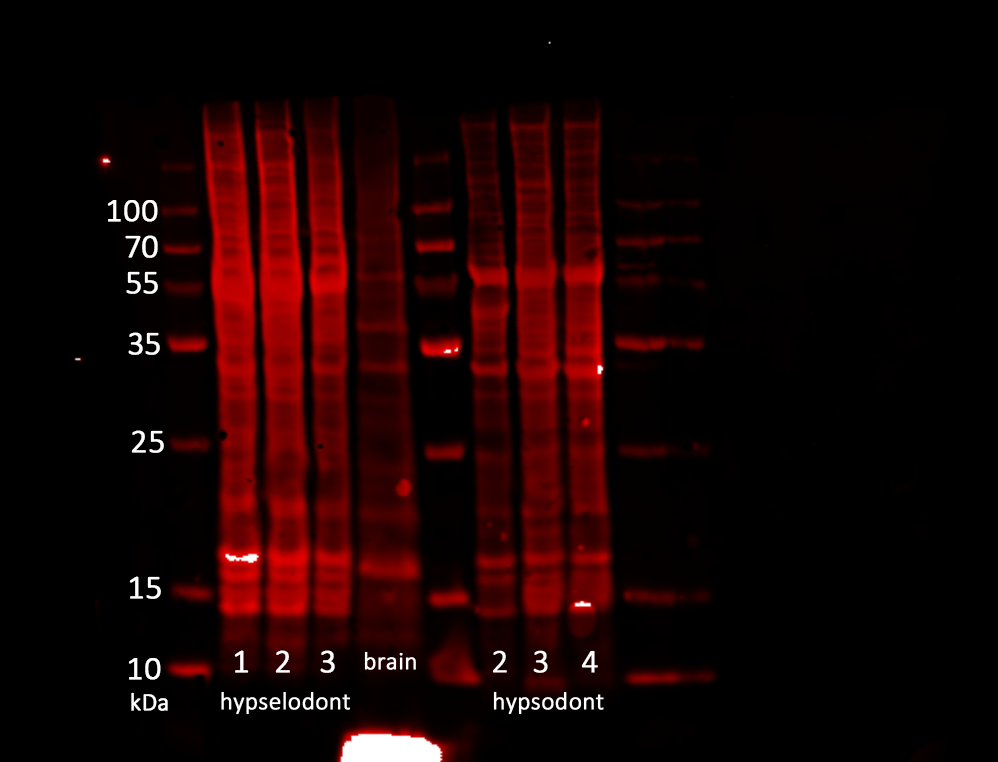

Supplement: Supplementary file 1 [file animals-16-00341-s001.zip › Figure S1_TP_700-channel_Fig6-5a-b.tif]

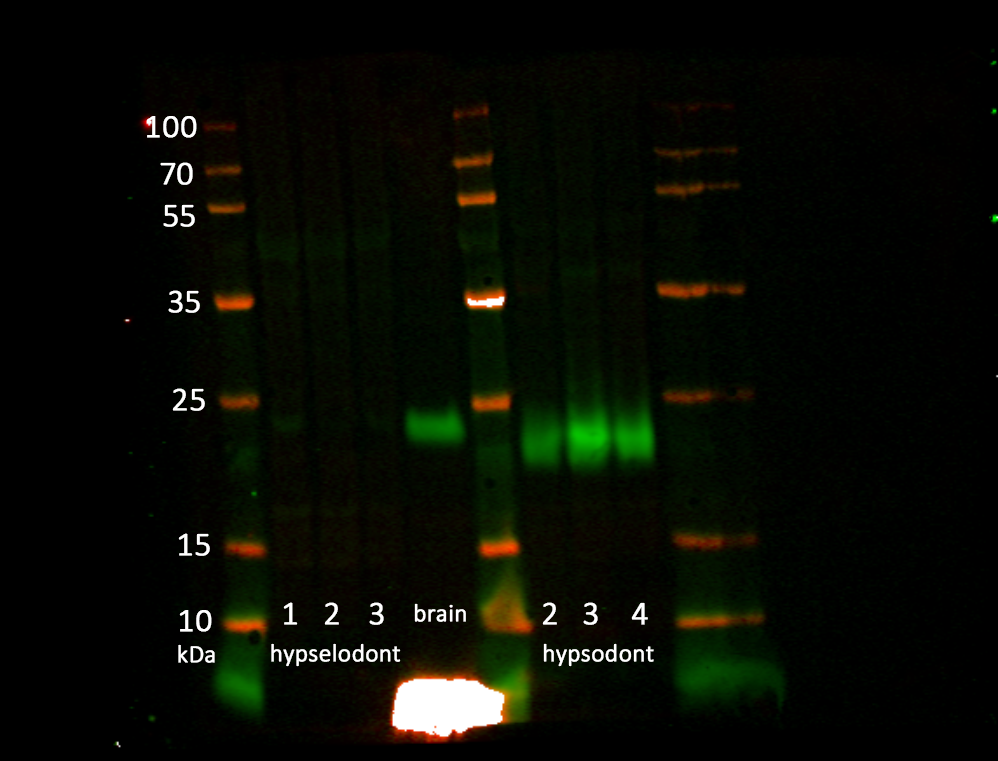

Supplement: Supplementary file 1 [file animals-16-00341-s001.zip › Figure S2_CD90_800-700-Fig6-5a-b.tif]

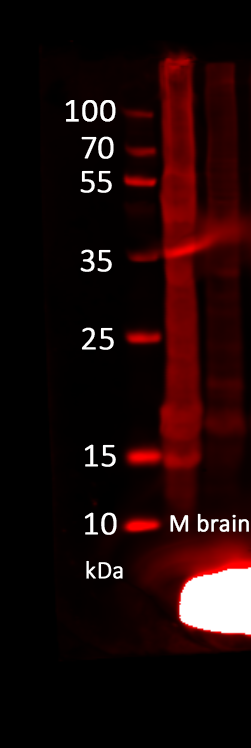

Supplement: Supplementary file 1 [file animals-16-00341-s001.zip › Figure S3_TP_700-channel_Fig6-5c.tif]

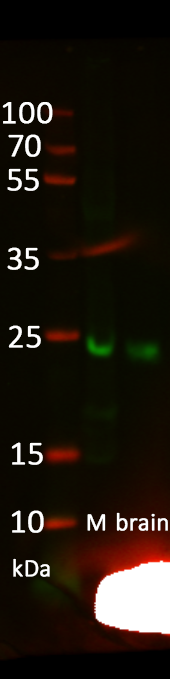

Supplement: Supplementary file 1 [file animals-16-00341-s001.zip › Figure S4_CD90_700-800-Fig6-5c.tif]
